# Identification of candidate genes associated with cell wall digestibility and eQTL (expression quantitative trait loci) analysis in a Flint × Flint maize recombinant inbred line population

**DOI:** 10.1186/1471-2164-8-22

**Published:** 2007-01-18

**Authors:** Chun Shi, Anna Uzarowska, Milena Ouzunova, Matthias Landbeck, Gerhard Wenzel, Thomas Lübberstedt

**Affiliations:** 1 Chair of Plant Breeding, Technical University of Munich, Am Hochanger 2, 85350 Freising, Germany; 2 KWS Saat AG, Grimsehlstrasse 31, 37555 Einbeck, Germany; 3 Department of Genetics and Biotechnology, Research Centre Flakkebjerg, 4200 Slagelse, Denmark

## Abstract

**Background:**

Cell-wall digestibility is the major target for improving the feeding value of forage maize. An understanding of the molecular basis for cell-wall digestibility is crucial towards breeding of highly digestible maize.

**Results:**

865 candidate ESTs for cell-wall digestibility were selected according to the analysis of expression profiles in 1) three sets of brown-midrib isogenic lines in the genetic background of inbreds 1332 (1332 and 1332 *bm3*), 5361 (5361 and 5361 *bm3*), and F2 (F2, F2 *bm1*, F2 *bm2*, and F2 *bm3*), 2) the contrasting extreme lines of FD (Flint × Dent, AS08 × AS 06), DD1 (Dent × Dent, AS11 × AS09), and DD2 (Dent × Dent, AS29 × AS30) mapping populations, and 3) two contrasting isogenic inbreds, AS20 and AS21. Out of those, 439 ESTs were assembled on our "Forage Quality Array", a small microarray specific for cell wall digestibility related experiments. Transcript profiles of 40 lines of a Flint × Flint population were monitored using the Forage Quality Array, which were contrasting for cell wall digestibility. Using *t*-tests (*p *< 0.01), the expression patterns of 102 ESTs were significantly different between high and low quality groups. Using interval mapping, eQTL (LOD ≥ 2.4) were detected for 20% (89 of 439) of the spotted ESTs. On average, these eQTL explained 39% of the transcription variation of the corresponding ESTs. Only 26% (23 of 89) ESTs detected a single eQTL. eQTL hotspots, containing greater than 5% of the total number of eQTL, were located in chromosomal bins 1.07, 1.12, 3.05, 8.03, and 9.04, respectively. Bin 3.05 was co-localized with a cell-wall digestibility related QTL cluster.

**Conclusion:**

102 candidate genes for cell-wall digestibility were validated by genetical genomics approach. Although the cDNA array highlights gene types (the tested gene and any close family members), trans-acting factors or metabolic bottlenecks seem to play the major role in controlling heritable variation of gene expression related to cell-wall digestibility, since no *in silico* mapped ESTs were in the same location as their own eQTL. Transcriptional variation was generally found to be oligogenic rather than monogenic inherited due to only 26% ESTs detected a single eQTL in the present study. One eQTL hotspot was co-localized with cell wall digestibility related QTL cluster on bins 3.05, implying that in this case the gene(s) underlying QTL and eQTL are identical. As the field of genetical genomics develops, it is expected to significantly improve our knowledge about complex traits, such as cell wall degradability. Comprehensive knowledge of the lignin pathway and cell wall biogenesis will allow plant breeders to choose the best genomic targets controlling these characters, for improving forage digestibility through genetic engineering or marker-assisted selection.

## Background

Lignin content is well known as a major factor affecting forage quality in maize. However, correlations between lignin content and forage quality can be variable according to the genetic background [1]. Moreover, breeding for a higher digestibility of maize involves also other, so far unknown mechanisms [1]. Correlations between maize whole plant digestibility and cell-wall (or stover) digestibility ranged from 0.60 to 0.96, with average values close to 0.80, whereas correlations between whole-plant digestibility and grain or ear content were close to 0.4 [2]. Therefore, cell-wall digestibility is the major target for improving the feeding value of forage maize. An understanding of the molecular basis for cell-wall digestibility is crucial towards breeding of highly digestible maize.

An important first step to elucidate the mechanisms underlying cell-wall degradability is to identify causative genome regions. Five major QTL clusters involved in cell-wall digestibility, located on chromosomal bins [3] 1.03, 3.05/06, 6.06, 8.05, and 9.02, were identified by several QTL analyses, but the genes underlying these QTL are not yet known [1]. A second, more recent approach is transcriptome analysis to simultaneously measure the expression of thousands of genes. In comparison with normal maize genotypes, brown-midrib (*bm*) mutants show a significantly reduced lignin content, altered lignin composition, and/or a significantly higher cell-wall digestibility [4]. Molecular mechanisms underlying cell wall digestibility in maize have been studied in three sets of maize brown-midrib isogenic lines in the genetic background of inbreds 1332 (1332 and 1332 *bm3*), 5361 (5361 and 5361 *bm3*), and F2 (F2, F2 *bm1*, F2 *bm2*, and F2 *bm3*) [5]. 53 ESTs were differentially expressed in all three isogenic *bm3 *comparisons, whereas 32 ESTs were consistently differentially expressed in different *bm *isogenic lines in F2 background. Moreover, gene expression studies can be conducted on phenotypically extreme lines from mapping populations. Replicate pools of extreme lines can be profiled independently, so that differences in gene expression will be specific to the differing pools. This strategy was recently used to identify candidate genes for drought response QTL in rice [6]. Thus, by synthesis of expression profiling data from *bm *mutants and extreme lines of a mapping population segregating for cell wall digestibility, it should be possible to identify candidate genes related to cell-wall degradability, and to construct a microarray enriched for candidate genes underlying cell wall digestibility. Transcriptome analysis using such microarrays would provide a fingerprint of cell-wall metabolism in maize.

Genetic and gene expression approaches have been joined in the concept of "genetical genomics" [7], which aims to detect eQTL (expression quantitative trait loci) controlling gene expression differences. Often, eQTL map to the genetic position of the respective gene itself, indicating that cis changes (within the gene) are responsible for the different levels of expression. In contrast, genes revealing (trans) eQTL at positions different from the genetic position of the respective gene are thought to be regulated by, e.g., trans-acting factors controlling their expression levels [8]. Detection of the master regulators, affecting expression levels of groups of genes, is a major feature of eQTL studies [9]. In plants, this strategy has been successfully applied to 76 maize lines in a F3 population [10] and 91 poplar lines in a backcross population [11], respectively. Altogether, combining expression profiling with genetic analysis could enrich our understanding of regulatory networks underlying cell-wall digestibility and assist plant breeders to choose the most relevant genomic targets for improvement of silage maize digestibility.

The objectives of our study were to 1) select candidate ESTs for cell-wall digestibility to establish a "Forage Quality Array", 2) identify ESTs differentially expressed between low and high digestible lines in a Flint × Flint mapping population, and 3) detect eQTL using the "genetical genomics" approach.

## Results

### Selection of candidate ESTs in association with cell wall digestibility

In order to identify genes in association with cell-wall digestibility in maize, three sources of genetic material were used, including 1) three sets of brown-midrib isogenic lines in the genetic background of inbreds 1332 (1332 and 1332 *bm3*), 5361 (5361 and 5361 *bm3*), and F2 (F2, F2 *bm1*, F2 *bm2*, and F2 *bm3*) [5], 2) the contrasting extreme lines of FD, DD1, and DD2 DH mapping populations, and 3) two isogenic inbreds, AS20 and AS21, significantly differing in cell wall digestibility. Two complementary approaches, SSH (suppression subtractive hybridization) and microarray-based expression profiling, were used to isolate and identify candidate genes in all comparisons. About 70% of the ESTs isolated by SSH were absent on the unigene microarray [5].

**Figure 1:**
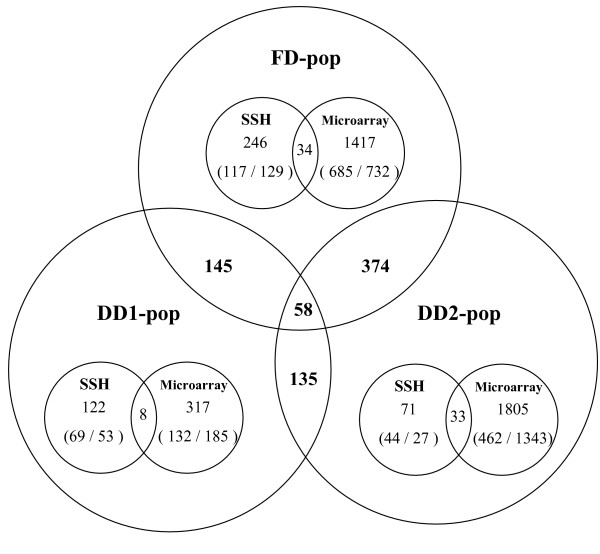
**Venn diagram for the summary of differentially expressed ESTs identified from FD (Flint × Dent, AS08 × AS 06), DD1 (Dent × Dent, AS11 × AS09), and DD2 (Dent × Dent, AS29 × AS30) mapping populations, respectively**. Total numbers of genes differentially expressed are indicated in respective circles. In parentheses, the first number indicates up-, the last number down-regulated genes. The number of genes simultaneously up- or down-regulated is given in intersections between circles.

The number of at least two-fold induced ESTs in stems from SSH and microarray analyses, respectively, were 246 and 1417 in FD-pop (Figure 1), 122 and 317 in DD1-pop, 71 and 1805 in DD2-pop, as well as 225 and 630 in AS20 vs. AS21 (Data not shown). In FD-pop, 34 ESTs were jointly differentially expressed both in SSH and microarray experiments, as well as 8, 33, and 31 ESTs in DD1-pop, DD2-pop, and AS20 vs. AS21, respectively. The numbers of genes simultaneously differentially expressed in pairs of mapping populations were 145 (between FD-pop and DD1-pop), 374 (between FD-pop and DD2-pop), and 135 (between DD1-pop and DD2-pop), respectively. 58 ESTs were consistently differentially expressed across the three mapping populations.

**Figure 2:**
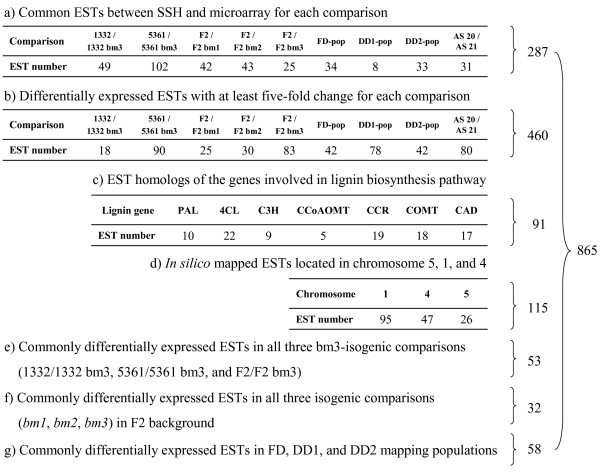
Selection of 865 candidate ESTs for cell wall digestibility based on seven selection criteria.

In total, 5460 distinct ESTs differentially expressed in one or multi- comparisons were identified. In order to verify the most interesting of those genes at low costs, a small microarray specific for cell wall digestibility (Forage Quality Array) was designed. The first selection criterion (Figure 2) covered jointly differentially expressed ESTs both in SSH and microarray experiments for each comparison (287 ESTs). Secondly, 460 ESTs were selected with a more than five-fold change in respective comparisons. Thirdly, 91 EST homologues of lignin-related genes, PAL, 4CL, C3H, CCoAOMT, CCR, COMT, and CAD, were spotted. Subsequently, 115 ESTs *in silico *mapping to chromosomes 1, 4, and 5 were chosen, since *bm1*, *bm2*, and *bm3 *map to these chromosomes. Finally, 53, 32, and 58 consistently differentially expressed ESTs in (i) all three bm3-isogenic comparisons (1332 vs. 1332 bm3, 5361 vs. 5361 bm3, and F2 vs. F2 bm3), (ii) all three isogenic comparisons in F2 background (F2 vs. F2 bm1, F2 vs. F2 bm2, and F2 vs. F2 bm3), and (iii) three mapping populations, respectively, were selected. According to the above mentioned criteria, 865 different candidate ESTs were identified towards production of the Forage Quality Array.

**Figure 3:**
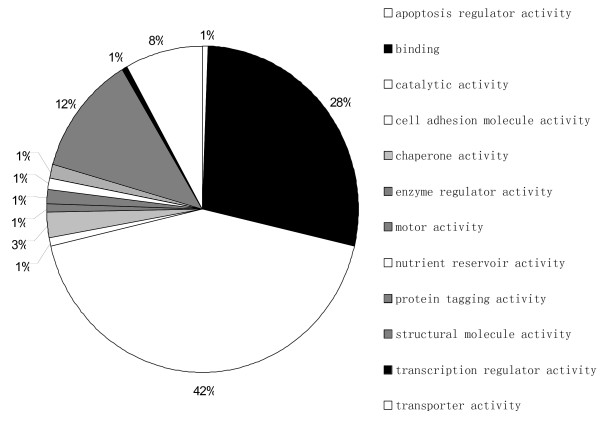
**Assignment of the 439 ESTs spotted on the “Forage quality array” to functional classes by GO vocabularies.**   Each EST was assigned with GO vocabularies according to the TIGR Maize Gene Index [12]. Unclassified ESTs (66%) were not taken into account.

Out of 865 candidate ESTs, 151 were not available for ordering, and for 275 ESTs it was not possible to obtain high-quality PCR products due to poor bacteria recovery, unspecific PCR amplification, etc. Finally, 439 ESTs were included on the Forage Quality Array. GO vocabularies were assigned according to the TIGR Maize Gene Index [12]. 66% of these ESTs (288 of 439) could not be functionally classified. Among 151 classified ESTs, the largest category was "catalytic activity" (42%) (Figure 3). Further ranking of classification categories was "binding" (28%), "structural molecule activity" (12%), and "transporter activity" (8%).

**Figure 4:**
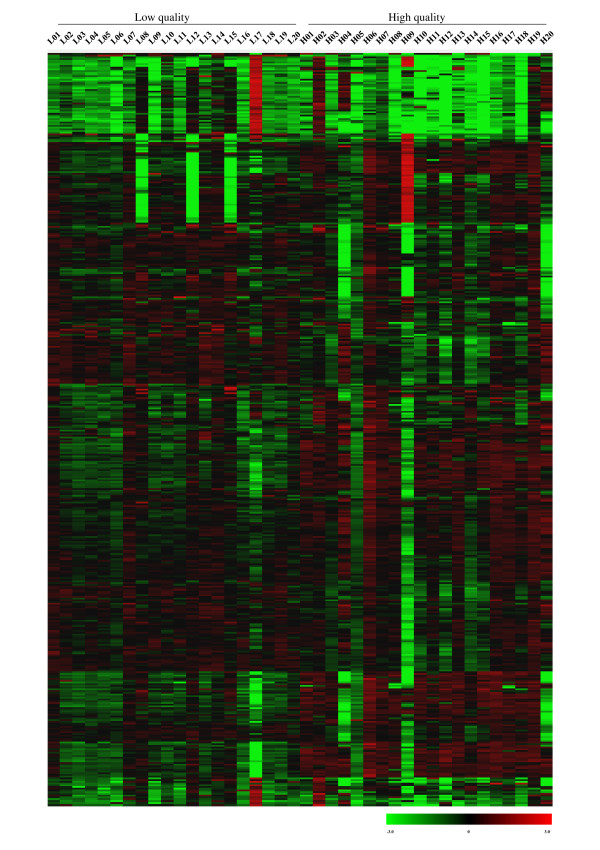
**The expression profiling of 439 ESTs in 2 × 20 recombinant inbred lines contrasting for dNDF**. The color saturation reflects the magnitude of the log2 expression ratio (Cy5/Cy3) for each transcript. Each row represents an EST. Red and green colors mark up-regulated and down-regulated ESTs in individual line, respectively. The color log2 scale is provided at the bottom of this figure.

Using hierarchical cluster analysis based on the EST tree, distinct expression patterns between high and low quality groups were found for most ESTs included on the Forage Quality Array (Figure 4). Using *t*-tests with a *p *value cutoff of 0.01, 102 ESTs were significantly different between the two groups of high and low quality lines, including 39 down- and 63 up-regulated ESTs in the high quality group, respectively (Table 1). Out of 39 down-regulated ESTs in the high quality group, 1, 8, and 5 ESTs were classified into "chaperone activity", "catalytic activity", and "binding", respectively, whereas 1, 11, 2, 1, and 4 ESTs of up-regulated ESTs were classified into "motor activity", "catalytic activity", "structural molecular activity", "transporter activity" and "binding", respectively. According to the current annotation in the TIGR Maize Gene Index [12], four ESTs encoded enzymes involved in lignin biosynthesis, CD970581 (Cinnamyl-alcohol dehydrogenase), CD973094 (Phenylalanine ammonia-lyase), AW120445 (Class III peroxidase 67 precursor) and CF243853 (Caffeoyl CoA 3-O-methyltransferase). In addition, AI622068 (Cellulose synthase-2) is a homologue encoding a cellulose biosynthesis related enzyme, and BM333894 (YABBY-like transcription factor) and BU093700 (transcription factor and jumonji family protein) are putative transcription factors.

### Genetical genomics analysis

40 extreme lines in the FF population with respect to dNDF were selected to represent two contrasting high and low quality groups (Table 2). The dNDF mean and SD of the low quality group was 52.34% and 2.12, and 62.30% and 1.32 in the high quality group.

**Figure 5:**
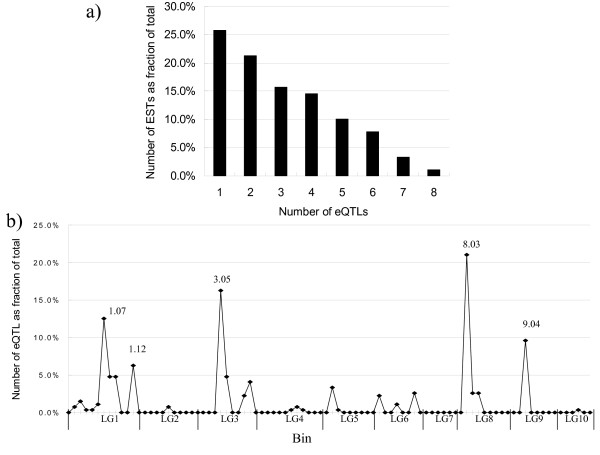
**Expression quantitative trait loci (eQTL) (LOD ≥ 2.4) distributions**. a) Distribution of numbers of eQTLs detected for corresponding EST; b) Genome-wide distribution of eQTLs according to the bins.

Using interval mapping, eQTL (LOD ≥ 2.4) were detected for 20% (89 of 439) of the tested ESTs, with a maximum LOD score of 5.2. On average, these eQTL explained 39% of the genetic variation of the corresponding EST expression profiles, ranging from 24.1% to 91.4%. The number of eQTL per EST varied from 1 to 8 with a mean of 3 (Figure 5a). For 63% (56 of 89) ESTs, one to three eQTL were found. For four ESTs more than 6 eQTL were detected. 39% (35 of 89) ESTs with eQTLs were significant for high and low pools as well.

**Figure 6:**
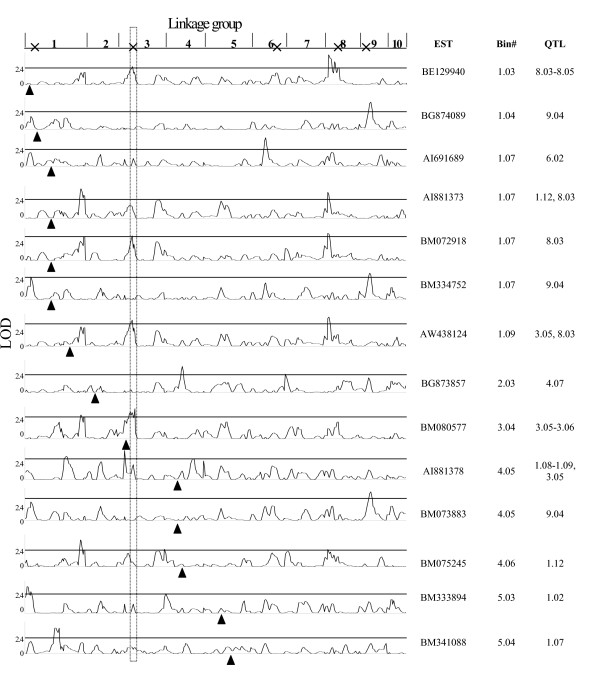
**LOD score profiles generated by interval mapping analysis of dNDF and expression levels of 14 ESTs in silico mapped among 89 ESTs resulting in eQTL**. The black triangles indicate the *in silico *mapping position for each EST. EST mapping information was extracted from the Maize GDB [13] and the IDP mapping project [27], according to map bins [3]. The *x *axis represents ten linkage groups of the Flint × Flint genetic map arranged end-to-end. The middle line represents LOD score threshold 2.4. The "× " indicates five major QTL clusters for cell wall digestibility on bins 1.03, 3.05/06, 6.06, 8.05, and 9.02 [1]. The dashed rectangle shows some eQTLs co-localizing with one major QTL cluster for cell wall digestibility on bin 3.05.

Maize genetic maps have been divided into 100 segments (=bins) of approximately 20 cM length [3]. In total, 271 eQTL were detected in 24 bins covering all chromosomes except for chromosome 7 (Figure 5b). The percentage of eQTL per bin was plotted against chromosomal bins (Figure 5). eQTL hotspots, containing more than 5% of the total number of eQTL, were detected on bins 1.07, 1.12, 3.05, 8.03, and 9.04. Out of those, bin 3.05 co-localized with one of five major QTL clusters for cell-wall digestibility (Figure 6), which were identified on bins 1.03, 3.05/06, 6.06, 8.05, and 9.02 [1].

Out of 89 ESTs resulting in eQTL, for 14 ESTs bin information from the Maize GDB [13] and map positions from Maize Genome Mapping project [14] (Figure 6) are known. For another 10 ESTs, only chromosomal but not bin assignment were provided from Maize Genome Mapping project. *In silico *mapping information indicate that the ESTs were located in various linkage groups, and generally not in the genetic location of the respective eQTL. AW438124 (nucleosome/chromatin assembly factor C), BM080577 (nodulin MtN3 family protein), and AI881378 (maize clone contig234 mRNA sequence) were co-localized with a cell-wall digestibility related QTL cluster on bin 3.05.

## Discussion

### Power of eQTL analysis

An essential first step in QTL analysis is to assess how many samples must be collected in order to achieve sufficient power to detect a hypothetical effect. eQTL mapping is different from QTL mapping of complex inherited traits, since we expect (and are mainly interested in) major eQTL due to 1) eQTL in cis (then most of the variation should be explained by this one eQTL), and 2) major regulatory eQTL (one or few) in trans. Thus, we expect a simpler inheritance of expression patterns of single ESTs with only one or few eQTL involved, each explaining a comparatively large percentage of the phenotypic variation, in contrast to "true" quantitative characters such as grain yield. Major eQTL should be detectable even with this very limited number of RILs. In addition, this requirement is particularly salient in eQTL analysis due to the high expenses of gene-expression analyses. In plants, both 76 F3 families in maize [10] and 91 BC1 families in poplar [11] were used in respective eQTL analysis. In this study, 40 RIL lines were used to detect eQTL in the FF population. Because of an increased heritability when using homozygous and homogeneous RIL lines as compared to segregating F2:3 families, an eQTL explaining 28% of the variation in RNA abundance among the F2 population would be detected in 99% of the experiments with 40 RI lines, in contrast to 94% of the experiments performed with 76 F3 families [9]. In addition, at least 73% of the experiments with 40 RIL lines should detect an eQTL explaining 18% of the phenotypic variance [9]. Since 79% (214 of 271) identified eQTL explained more than 28% of the transcription variation of the corresponding ESTs and minimum eQTL explaining 24.1% transcription variation, 40 extreme lines should be sufficient to detect major eQTL. However, the explained phenotypic variance by individual eQTL is very likely overestimated due to the small population size [15].

### Genetic architecture of transcription

Genetical genomics permits quantitative assessment of the proportion of gene loci (ESTs) displaying co-segregation with the respective eQTL (cis-eQTL), relative to those that produce unlinked trans-eQTL [13]. In maize ear leaf, 34% (6481 of 18,805) differentially expressed genes produced cis-eQTL with LOD >3.0 [10]. In contrast, no *in silico *mapped ESTs were in the same location as their own eQTL in this study (Figure 6). Accordingly, no key genes involved in lignin biosynthesis co-localized with cis-eQTL in poplar [11]. Therefore, trans-acting factors could play a major role in regulating heritable variation of gene expression in cell wall digestibility.

Only 26% (23 of 89) ESTs detected a single eQTL in the present study. For the remaining 74%, two or more eQTL were found (Figure 5). A similar distribution of eQTL was found in yeast [16]. Only 3% transcripts were consistent with single locus inheritance, 18% suggested control by two loci, and >50% required at least five loci under an additive model. Thus, transcriptional variation was generally found to be oligogenic rather than monogenic inherited.

A common feature of eQTL studies is the detection of "hotspots" or hubs of trans-acting eQTL: chromosomal regions that affect the expression of a much larger number of genes than expected by chance [9]. eQTL hotspots, containing more than 5% of the total number of eQTL, were found on bins 1.07, 1.12, 3.05, 8.03, and 9.04 (Figure 5). The strong clustering in hubs of eQTL reflects highly correlated expression levels of many gene transcripts in association with cell-wall digestibility. The five hotspots could contain important transcript factors for cell wall digestibility. However, no obvious transcription factors were found among those genes currently mapped to these bin regions [13]. Similarly, eQTL identified in yeast frequently resulted from genetic variants that altered activity of a signaling or metabolic pathway without directly altering gene transcription [17]. 4CL2, F5H, and CCR1 genes involved in lignin biosynthesis map to bin 1.07 [1]. These genes are candidates for the eQTL found in the same genome region due to potential metabolic feedback mechanisms acting through the same enzyme(s) in lignin biosynthesis. However, due to the limited understanding of genetic and physiological control of gene expression so far, any conclusions with regard to hotspots for gene regulation should be interpreted with caution [9]. Kirst et al. [11] found that two eQTL hubs for lignin-related genes co-localized with growth QTL on linkage groups 4 and 9 in poplar. One eQTL hotspot was co-localized with cell wall digestibility related QTL cluster [1] on bins 3.05 (Figure 6), implying that in this case the gene(s) underlying QTL and eQTL are identical.

### Molecular mechanisms underlying cell-wall digestibility

Lignin restricts the degradation of structural polysaccharides by hydrolytic enzymes, thereby limiting the bioconversion of forages into animal products [18]. Out of 102 significantly differentially expressed ESTs between high and low quality groups (Table 2), four ESTs encode enzymes involved in lignin biosynthesis, CD970581 (Cinnamyl-alcohol dehydrogenase), CD973094 (Phenylalanine ammonia-lyase), BE051493 (4-coumarate-CoA ligase-like protein), and CF243853 (Caffeoyl CoA 3-O-methyltransferase). In agreement with the expectation of low levels of lignin, CD970581 and CD973094 were down-regulated in lines with high forage quality. However, BE051493 and CF243853 were up-regulated, which might be due to the complexity of cell wall biosynthesis. The associations between one lignin characteristic (e.g., lignin composition) and cell wall degradability can be confounded and correlated to concurrent changes in other lignin properties (e.g., lignin cross-linking) that influence cell wall degradability [18]. Since reduced cellulose synthesis invoked lignification and defense responses in *Arabidopsis *[19], repressed ESTs encoding cellulose synthesis enzymes, such as AI622068 (Cellulose synthase-2) (Table 2), could activate lignin synthesis in low quality lines. In addition, four regulatory genes, AI734769 (Class III HD-Zip protein 4), BM080754(Translational initiation factor eIF-4A), BM334752 (Zinc finger protein family-like), and BM333894 (YABBY-like transcription factor), were down-regulated in lines with high forage quality, whereas one regulatory gene, BU093700 (transcription factor and jumonji family protein) was up-regulated (Table 2). These regulatory genes might control cell wall digestibility spatially and temporally among the two groups of lines in the FF population. Mapping of these genes will reveal, whether they colocalize with eQTL (clusters) identified in this study. Furthermore, there are a number of genes, where a connection to digestibility is unclear, some of which might be false positives.

Using cellular UV-microspectrophotometry, Shi et al. [5] demonstrated that lignin content represented by the absorbance values within the cells of sclerenchyma fiber tissue was decreasing from the periphery to the center. Lignin content was lowest in S2 (secondary cell wall) of parenchyma cells, followed by S2, CML (compound middle lamella) and CC (cell corner) of sclerenchyma fibers. Each cell type in the stem likely expresses a unique transcriptome. Transcriptome analysis using complete stems provides average gene expression levels integrated over all cell types. In order to further understand the molecular basis of cell wall degradability, new methods, for instance, laser-capture microdissection [20], are needed for efficient isolation of CC of sclerenchyma fiber from different time points to further validate 439 candidate genes.

### Application of genetical genomics to plant breeding

The use of functional genomics is contributing to many aspects of QTL analysis and cloning [21]. To date, most plant QTL have been cloned using a positional cloning approach following identification in experimental crosses. Transcriptional profiling can quickly provide a list of differentially expressed genes between contrasting QTL genotypes. Subsequently, those genes functionally related to the target trait and mapping to the QTL region can be selected as candidates. Unfortunately, the number of QTL cloned to date in plants is too small to test the validity of this approach. If the difference in gene expression level between alleles is too low (approximately twofold), the candidate genes cannot be identified using standard microarray-based transcriptome analysis [21]. Less than 15% of ESTs were induced more than two-fold by microarray analyses in FD-pop (12%, 1,417 of 11,827) (Figure 1), DD1-pop (3%, 317 of 11,827) and DD2-pop (15%, 1,805 of 11,827), respectively. A genetical genomics approach might be better suited for detecting differences in gene expression. 23% of the ESTs on the Forage Quality Array (102 of 439) were significantly different between low and high digestible (dNDF) RILs. However, this higher proportion of significantly differentially expressed genes identified by the genetical genomics approach is most likely due to successful selection of candidates by the criteria outlined above (Figure 2).

Since most of the important agronomic traits are complex inherited traits, transcript abundance may act as an intermediate phenotype between genomic DNA sequence variation and complex traits. Integration of genome-wide expression profiling with linkage analysis is a new approach to identifying genes underlying complex traits. Hubner et al. [22] has demonstrated large-scale identification of positional candidates and regulatory pathways for previously mapped physiological QTL by genetical genomics approach in the rat. Cis-acting eQTL are good candidates for physiological QTL because they show line-specific differences in gene expression that are under the control of DNA sequence variants in or close to the gene itself [23]. Similarly, eQTL identified in an intercross of inbred C57BL/6J and DBA/2J mice could accurately identify transcripts in which expression was regulated by cis-acting polymorphisms [24]. Cis-acting eQTL might be detected by additional EST mapping or indirectly through the ongoing maize genome sequencing project, since so far only 24 of 89 ESTs resulting in eQTL were *in silico *mapped. Trans-acting eQTL represent loci that influence expression of genes or transcripts remote from the eQTL itself. Coincidental mapping of trans-acting eQTLs for multiple transcripts to the same chromosomal location, as observed on bin 3.05 (Figure 6), may represent a shared regulatory transcriptional control mechanism by a single gene at the eQTL. The locations of trans-acting eQTL in relation to physiological QTL, together with the locations of cis-acting eQTL, may point to genes and regulatory pathways underlying individual cell wall digestibility related QTL.

However, the scope of genetic analysis of gene expression also presents enormous technical and analytical challenges. Extraordinarily large number of comparisons were involved in a genome-wide linkage scan for several hundreds or thousands of transcripts, thus technical and experimental design issues need to be addressed to handle the large data sets that are being generated, and new statistical tools are still being evaluated [25]. As the field of genetical genomics develops, it is expected to significantly improve our knowledge about complex traits, such as cell wall degradability [8]. Therefore, in the future, it is conceivable that QTL cloning will increasingly rely on candidate gene information. Comprehensive knowledge of the lignin pathway and cell wall biogenesis will allow plant breeders to choose the best genomic targets controlling these characters, for improving forage digestibility through genetic engineering or marker-assisted selection.

## Conclusion

102 candidate genes for cell-wall digestibility were validated by genetical genomics approach. Although the cDNA array highlights gene types (the tested gene and any close family members), trans-acting factors or metabolic bottlenecks seem to play the major role in controlling heritable variation of gene expression related to cell-wall digestibility, since no *in silico *mapped ESTs were in the same location as their own eQTL. Transcriptional variation was generally found to be oligogenic rather than monogenic inherited due to only 26% ESTs detected a single eQTL in the present study. One eQTL hotspot was co-localized with cell wall digestibility related QTL cluster on bins 3.05, implying that in this case the gene(s) underlying QTL and eQTL are identical. As the field of genetical genomics develops, it is expected to significantly improve our knowledge about complex traits, such as cell wall degradability. Comprehensive knowledge of the lignin pathway and cell wall biogenesis will allow plant breeders to choose the best genomic targets controlling these characters, for improving forage digestibility through genetic engineering or marker-assisted selection.

## Methods

### Plant materials

AS20, AS21, recombinant inbred lines (RILs), and doubled haploid (DH) lines were obtained from KWS Saat AG, including twenty highly digestible and twenty low digestible lines each in four mapping populations: FD (Flint × Dent DH population, AS08 × AS 06), DD1 (Dent × Dent DH population, AS11 × AS09), DD2 (Dent × Dent DH population, AS29 × AS30), and FF (Flint × Flint RIL population, AS18 × AS07) RIL mapping populations, respectively. These lines can be obtained from KWS Saat AG for non-commercial research purposes. All plants were grown and maintained in the greenhouse under a 12 h photoperiod at 23°C and 50% relative humidity. Stems were harvested 5 weeks after germination, since the highest level of *COMT *expression was observed in stems 5 and 7 weeks after germination [5]. For biological replication, two independent sets of three plants per set were harvested for AS20, AS21, and every line in FD, DD1, and DD2 populations, respectively, and one set of ten plants per line in the FF population. Since the maize stalk is small and covered by leaf sheath at early vegetative stages, the stem fraction investigated in this study is a mixture of stalk and leaf sheath [26]. Total RNA was extracted from the stems of each set of each line using TRIzol reagent (Invitrogen, Carlsbad, CA, USA).

### Construction and hybridisation of Forage Quality Array

The macroarrays containing SSH clones were derived from the studies on three sets of maize brown-midrib isogenic lines in the genetic background of inbreds 1332 (1332 and 1332 *bm3*), 5361 (5361 and 5361 *bm3*), and F2 (F2, F2 *bm1*, F2 *bm2*, and F2 *bm3*) [5]. In total, 2688 clones were spotted in duplicate on each macroarray. These clones were randomly picked from five SSH libraries. For two stem pairs (5361 vs. 5361 *bm3*; 1332 vs. 1332 *bm3*) subtractions were conducted in both directions. For the root pair 1332 versus 1332 *bm3 *the hybridization was performed only in forward direction. Microarray hybridization data were evaluated by the SpotReport™ Alien™ cDNA Array Validation System (Stratagene, La Jolla, CA, USA), including positive, negative, and 10 spiking controls. Out of 2688 clones, 1,401 clones, ranging in length from 74 to 989 bp, were sequenced by MWG (Ebersberg, Munich, Germany) and clustered into 765 ESTs. Their sequences were analyzed in the same way as microarray-ESTs.

In the FD, DD1, and DD2 populations, the total RNA representing high and low forage quality DH lines was pooled from the total RNA of those twenty lines in each of the populations with the highest or lowest digestibility (DNDF), respectively. The pooled total RNA and the total RNA of AS20 and AS21 were labeled with ^32^P and hybridized to different arrays using the Strip-EZ RT kit (Ambion, Austin, TX, USA). Hybridization signals were detected by the Storm 860 imaging system (Amersham) with a resolution of 50 μm. After scanning, labeled cDNA probes were completely stripped from the arrays using the Strip-EZ system (Ambion). Each array was hybridized four times with four replicates per line, including two biological replications and two labeling replications in each biological replica.

Maize unigene-microarrays were provided by the laboratory of Prof. Schnable (Iowa University, USA) and contained 11,827 maize ESTs [27]. Poly (A)+ RNA was isolated from the same total RNA for probe preparation in the SSH approach via Dynabeads^® ^Oligo(dT)25 (Dynal biotech, Oslo, Norway). According to TIGR Microarray Protocols [28], each mRNA sample was indirectly labeled with Cy3 or Cy5 (Amersham Pharmacia, Piscataway, NJ, USA) and hybridized with maize unigene-microarrays. Fluorescence signals were detected using the arrayWoRx^® ^Biochip Reader (Applied Precision, Issaquah, WA, USA). For high vs. low digestibility lines in FD, DD1, and DD2 populations as well as AS20 vs. AS21, four replications including two biological and two dye-swap replications within each biological replication were conducted. Thus, four maize gene chips were used in each comparison.

The raw images obtained from Storm 860 imaging system or arrayWoRx^® ^Biochip Reader were imported into ArrayVision 8.0 (Imaging Research, St. Catharines, Ontario, Canada) for spot detection and quantification of hybridization signals. Raw data exported from ArrayVision 8.0 were imported into Excel and converted to TIGR Array Viewer (TAV) format files. Data were normalized using intensity-dependent local regression (Lowess) implemented in the Microarray Data Analysis System (MIDAS) [29]. All calculated gene expression ratios were log 2-transformed and averaged over four replicates in each comparison. Differentially expressed ESTs at the 95% confidence level were determined using intensity-dependent Z-scores (with Z = 1.96) as implemented in MIDAS and the union of all genes identified in each comparison was considered significant in this experiment. Each EST was annotated and assigned with GO vocabularies according to the TIGR Maize Gene Index [12]. The mapped ESTs contain bin information from the Maize GDB [13] and the IDP mapping project [27].

Among 865 candidate ESTs for cell wall digestibility, 208 ESTs were ordered from the Iowa Schnable Lab [30] and 506 ESTs from the Arizona BAC/EST resource center [31]. From the stab cultures of these ESTs, plasmid minipreps were conducted by use of the R.E.A.L. Prep 96 Plasmid Kit (Qiagene AG, Germany). Using the plasmids of each EST as template, two independent re-amplification (100 μl) reactions were performed and pooled to reduce the effects of variation in PCR efficiency. Pooled PCR products were concentrated from 200 μl to approximately 25 μl using MultiScreen-PCR plates (Millipore, Billerica, MA, USA). Due to poor bacteria recovery and unspecific PCR amplification, high quality PCR products were derived from only 439 ESTs and spotted on Nexterion^® ^Slide A+ glass slides (SCHOTT Jenaer Glas GmbH, Germany) in a triple pattern using a QarrayMini spotter (Genetix GmbH, Germany). The total RNA of each line in the FF population was labeled and hybridized in the same way as the total RNA of pooled lines in the other three populations. Using loop design [32], each Forage Quality Array was hybridized with two contrasting lines (Table 1) labeled with different dyes. In total, 40 Forage Quality Arrays were used and each line was employed with dye-swap replication.

### Data analysis of the "genetical genomics" experiment

The raw images of Forage Quality Arrays obtained from a GeneTAC™ UC4 Microarray Scanner (Genomics Solutions Ltd., USA) were imported into ArrayVision 8.0 for spot detection and quantification of hybridization signals. In ArrayVision 8.0, Lowess normalization was implemented to remove dye effects. The transcript level of each EST was represented by the average over six measures, including dye swap replication and three in-slide replications within either Cy3 or Cy5 replications.

The dataset of all 40 lines in the FF population were imported in Multiexperiment Viewer (MeV) [29]. "Hierarchical cluster analysis" [33] was conducted to discover similar expression patterns across 439 ESTs, and "between subject t-tests (*p *< 0.05)" [34] were utilized to identify candidate genes differentially expressed between high and low digestible lines.

A complete linkage map was constructed by KWS Saat AG with 156 loci using 270 lines, including forty extreme lines, in FF population. Low quality lines have all unfavorable QTL/alleles for dNDF, and high quality all favorable QTL/alleles for dNDF. The same mapping population was used for both QTL (Krützfeldt et al., in preparation) and eQTL mapping. Five dNDF QTLs were identified, and the positions of the QTL were in good agreement with previous QTL studies [1]. Identification of QTL for gene expression traits was performed based on the 40 extreme lines using interval mapping implemented in MapQTL [35]. An empirical LOD score threshold of 2.4 was determined by permutation testing [36,37] and adopted from Kirst et al. [11].

Original microarray expression data presented in this manuscript are available through ArrayExpress [38] with accession number E-MEXP-253.

## Authors' contributions

CS carried out the most of experiments, performed the data analysis, and drafted the manuscript. AU carried out the hybridization of Forage Quality Arrays. MO conceived of the study and participated in its coordination. ML participated in the design of the study. GW conceived of the study and assisted CS with the experiments. TL conceived of the study, participated in its design, and drafted the manuscript. All authors read and approved the final manuscript.

## Figures and Tables

**Table 1:** Differentially expressed (p < 0.01) ESTs (102) between low and high digestible (dNDF) recombinant inbred lines.

**GA**^a^	**Tentative annotation**^b^	**Similarity**^b^	**Transcript level**			
			
			**Low quality**		**High quality**	
			
			**Mean**	**SD**	**Mean**	**SD**
***Down-regulated in lines with high dNDF content***						
*Chaperone activity*						
AI948333	UP|TBB6_MAIZE Tubulin beta-6 chain	100%	0.13	0.06	0.10	0.16
*Catalytic activity*						
AI666096	UP|SUS2_MAIZE Sucrose synthase 2	100%	0.22	0.07	0.17	0.07
AI881378	Zea mays clone Contig234 mRNA sequence		1.26	0.47	0.83	0.26
AW424679	Zea mays clone EL01N0513E01.d mRNA sequence		3.37	1.02	2.69	0.70
BG842237	UP|Q6ZL43_ORYSA Histone H2A	100%	1.63	0.55	1.25	0.48
BM073983	UP|Q9SXV0_ORYSA Cytochrome c oxidase subunit 6b-1	44%	2.96	1.06	1.92	0.68
CD970581	PIR|T02767|T02767 cinnamyl-alcohol dehydrogenase – maize {Zea mays}	100%	2.12	0.72	1.31	0.65
CD973094	UP|Q7M1Q5_ORYSA Phenylalanine ammonia-lyase	96%	1.91	0.68	1.73	0.60
CF028668	UP|Q9SLP6_MAIZE Ferredoxin	100%	3.46	1.10	2.79	0.88
*Binding*						
AI734769	UP|Q5D1M3_POPTR Class III HD-Zip protein 4	21%	0.20	0.08	0.14	0.19
AW042324	UP|Q9XEY1_TOBAC Nt-iaa2.3 deduced protein	60%	0.34	0.12	0.24	0.08
BG841395	UP|Q8RY51_ORYSA Glucose-6-phosphate dehydrogenase	100%	1.96	0.60	1.45	0.58
BM080754	UP|O24449_MAIZE Translational initiation factor eIF-4A	100%	0.76	0.21	0.38	0.39
BM334752	UP|Q7EYM0_ORYSA Zinc finger protein family-like	98%	3.52	1.05	2.91	0.93
*Unclassified*						
AI622221	PRF|NP_191537 expressed protein {Arabidopsis thaliana}	42%	1.00	0.33	0.68	0.20
AI622354	UP|Q8GTX9_ARATH Cell cycle control crn (Crooked neck) protein-like	17%	0.81	0.33	0.43	0.17
AI666222	UP|Q6CWV7_KLULA Kluyveromyces lactis strain NRRL Y-1140	5%	1.73	0.62	1.12	0.34
AI691363	GB|AF127565 ubiquitin-protein ligase 2 {Arabidopsis thaliana}	3%	0.37	0.14	0.23	0.08
AI692057	UP|Q6K8U3_ORYSA Leucine-rich repeat-like protein	29%	0.80	0.30	0.59	0.21
AI737226	UP|Q75GJ2_ORYSA Expressed protein (With alternative splicing)	89%	1.04	0.38	0.74	0.30
AI834139	UP|Q6J9V5_MAIZE Cf2-like protein	3%	0.28	0.12	0.16	0.07
AI834361	UP|C85A1_ORYSA Cytochrome P450 85A1 (C6-oxidase) (Dwarf protein)	10%	1.09	0.38	0.68	0.26
AI855219			0.40	0.15	0.31	0.15
AI947947	UP|Q6TY49_HYDMC Reductase 1	21%	0.57	0.18	0.46	0.23
AW018225			1.86	0.75	1.55	0.61
AW155860	UP|Q5WAM8_BACSK DNA-directed RNA polymerase	6%	1.58	0.40	1.24	0.45
AW400279	Zea mays clone EL01N0325H04.d mRNA sequence		2.18	0.69	1.88	0.48
AW400378	UP|Q9FU27_ORYSA CCCH-type zinc finger protein-like	40%	0.46	0.17	0.33	0.18
AW438085	PRF|NP_567701 expressed protein {Arabidopsis thaliana}	55%	0.98	0.47	0.41	0.27
AW787698	UP|Q94FN1_LOTJA Phosphatidylinositol transfer-like protein III	29%	3.68	1.21	2.97	0.87
BG837856	UP|Q9ZPI2_9BRAS Delta-12 desaturase	46%	2.41	0.73	1.64	0.54
BM333894	UP|Q6SS00_ANTMA YABBY-like transcription factor GRAMINIFOLIA	23%	0.53	0.20	0.36	0.15
BM334482			2.89	0.83	2.17	0.67
BM349452	PRF|NP_850290 expressed protein {Arabidopsis thaliana}	28%	0.78	0.55	0.09	0.08
BM350803	UP|Q9PF60_XYLFA Endo-1, 4-beta-glucanase	16%	0.85	0.54	0.42	0.15
BM500212	UP|Q9LQS9_ARATH T4O12.9	39%	0.61	0.33	0.18	0.14
CB350592			0.44	0.18	0.29	0.19
CF005238	UP|Q9SBX4_MAIZE Proline-rich protein precursor	51%	3.06	1.09	2.11	0.38
CF062028	UP|UPTG_MAIZE (P80607) Alpha-1, 4-glucan-protein synthase	100%	1.13	0.47	0.97	0.48
***Up-regulated in lines with high dNDF content***						
*Motor activity*						
AW065585	UP|Q9FUS4_SETIT Actin	100%	0.38	0.23	0.69	0.26
*Catalytic activity*						
AI622068	UP|Q9LLI8_MAIZE Cellulose synthase-2	10%	0.24	0.09	0.44	0.16
AI737885	UP|O60585_HUMAN Ser/Arg-related nuclear matrix protein	4%	0.94	0.31	1.85	0.77
AW927815	UP|TRXF_PEA (P29450) Thioredoxin F-type, chloroplast precursor	59%	0.24	0.11	0.48	0.20
BE050716	Zea mays clone EL01N0551B03.d mRNA sequence		0.08	0.03	0.12	0.06
BE129940	UP|PEPCK_MAIZE (Q9SLZ0) PEP carboxykinase	100%	0.42	0.15	0.92	0.37
BM334031	UP|HMT2_MAIZE (Q9FUM9) Homocysteine S-methyltransferase 2	100%	0.32	0.13	0.70	0.36
BM350652	UP|Q45W77_ARAHY Ubiquitin-conjugating enzyme 1	100%	0.68	0.28	1.40	0.60
CB617280	Zea mays clone EL01N0531D09.c mRNA sequence		0.14	0.05	0.29	0.16
CD972065	UP|Q8L7Y9_ARATH Phosphatidylglycerol specific phospholipase C	92%	0.02	0.04	0.05	0.16
CF030921	UP|Q9FNU9_DACGL Mitochondrial processing peptidase alpha-chain	100%	0.31	0.13	0.80	0.39
CF243853	UP|Q7X6T0_MAIZE Caffeoyl CoA 3-O-methyltransferase	100%	0.03	0.06	0.06	0.20
*Structural molecule activity*						
AI691512	UP|TBA2_MAIZE Tubulin alpha-2 chain	100%	0.41	0.23	0.70	0.24
AI920382	Zea mays clone Contig900.F mRNA sequence		0.38	0.14	0.66	0.23
*Transporter activity*						
AI621754	UP|Q9LSD4_ARATH	89%	0.68	0.49	1.38	0.42
*Binding*						
AW400061	UP|METK_ORYSA S-adenosylmethionine synthetase 1	100%	0.16	0.06	0.43	0.19
AW438124	UP|Q8W514_MAIZE MSI type nucleosome/chromatin assembly factor C	100%	0.18	0.07	0.35	0.14
BM078288	PRF|NP_200425 KH domain-containing protein	73%	0.11	0.04	0.24	0.13
BM381996	UP|O22470_ORYSA GDP dissociation inhibitor protein OsGDI1	80%	0.07	0.03	0.15	0.08
*Unclassified*						
AI622159	UP|O75833_HUMAN UTF1	5%	0.03	0.05	0.08	0.29
AI673886	UP|O82347_ARATH Expressed protein (At2g46220/T3F17.13)	61%	0.02	0.03	0.04	0.15
AI691788	GB|BT010134 At2g02560 {Arabidopsis thaliana}	50%	0.50	0.17	1.29	0.50
AI714870	UP|Q7XVJ6_ORYSA OJ000126_13.12 protein	89%	0.13	0.06	0.22	0.09
AI715073	UP|Q4EVD5_LISMO ATP-dependent RNA helicase DeaD	4%	0.43	0.22	1.14	0.42
AI857233	Zea mays clone EL01N0442A09.c mRNA sequence		0.06	0.02	0.12	0.10
AI881373	UP|O24293_PEA Chloroplast inner envelope protein, 110 kD precursor	32%	0.21	0.07	0.42	0.16
AI901469	UP|Q8GUG5_ARATH Threonine dehydratase/deaminase (OMR1)	27%	0.14	0.05	0.28	0.11
AI901508	UP|Q7TFG7_RHCM6 Rh175	11%	0.09	0.07	0.27	0.38
AI920330	UP|Q6I683_ORYSA UDP-glucuronic acid decarboxylase	91%	0.59	0.22	0.89	0.36
AI948303			0.96	0.31	1.67	0.61
AI964683	UP|NRTN_HUMAN (Q99748) Neurturin precursor	7%	0.59	0.16	0.94	0.37
AW065798	UP|RL10_MAIZE 60S ribosomal protein L10	39%	0.26	0.07	0.38	0.13
AW091349	UP|Q4NPG7_9DELT PE-PGRS family protein	3%	1.09	0.31	1.47	0.49
AW120445	UP|Q5U1M6_ORYSA Class III peroxidase 67 precursor	53%	2.06	0.73	3.42	1.15
AW146725	PRF|NP_192621 expressed protein {Arabidopsis thaliana}	60%	0.31	0.09	0.48	0.17
AW330680	UP|Q8L7U7_ARATH AT4g39670/T19P19_60	76%	0.17	0.05	0.36	0.16
AW331016			0.36	0.12	0.78	0.33
AW331105	PRF|NP_189346 expressed protein {Arabidopsis thaliana}	77%	0.19	0.08	0.47	0.32
AW331480	UP|Q6ILL4_DROME HDC09080	8%	0.15	0.07	0.29	0.12
AW400073	UP|Q9FRX0_MAIZE Bundle sheath cell specific protein 1	100%	0.14	0.04	0.27	0.12
AW438246	PRF|NP_197590 expressed protein {Arabidopsis thaliana}	20%	0.26	0.13	0.45	0.20
BE051493	UP|Q9M0X9_ARATH 4-coumarate-CoA ligase-like protein	34%	0.29	0.12	0.70	0.32
BE129888	UP|Q6K702_ORYSA MATE efflux protein-like	56%	0.03	0.05	0.10	0.27
BF728781	UP|Q9XEI4_MAIZE Unconventional myosin heavy chain	10%	0.31	0.12	0.70	0.31
BG321060	PRF|NP_567470 expressed protein {Arabidopsis thaliana}	23%	0.08	0.05	0.16	0.09
BG842356	UP|Q8L6I1_ORYSA Plasma membrane H+ ATPase	47%	0.09	0.06	0.23	0.13
BG842835	PRF|NP_195843 haloacid dehalogenase-like hydrolase family protein	91%	0.12	0.04	0.30	0.15
BM073617	UP|Q56WY6_ARATH Serine protease like protein	65%	0.48	0.22	1.12	0.43
BM080336	UP|Q9FZ91_ARATH F3H9.17 protein (Sulfotransferase family protein)	7%	0.38	0.18	0.64	0.30
BM080577	PRF|NP_193327 nodulin MtN3 family protein {Arabidopsis thaliana}	70%	0.74	0.22	1.52	0.55
BM350621	UP|Q4SGI8_TETNG Chromosome 3 SCAF14593	5%	0.29	0.09	0.78	0.36
BM382642	UP|PSBQ1_MAIZE (Q41048) Oxygen-evolving enhancer protein 3–1	100%	0.23	0.08	0.39	0.22
BQ279795	Zea mays clone Contig815.F mRNA sequence		0.33	0.15	0.78	0.35
BU093700	PRF|NP_680116 transcription factor jumonji family protein	7%	0.44	0.14	0.87	0.40
CB605303	UP|Q56WY6_ARATH Serine protease like protein	65%	0.17	0.07	0.35	0.16
CB815513	PRF|NP_850290 expressed protein {Arabidopsis thaliana}	28%	0.11	0.04	0.25	0.11
CD446744	Zea mays clone EL01T0206E01.c mRNA sequence		0.15	0.06	0.27	0.12
CD972441	UP|Q9XE75_SORBI Patatin-like protein	100%	0.24	0.10	0.42	0.19
CD984112	PRF|NP_174335 SIT4 phosphatase-associated family protein	9%	0.36	0.15	0.68	0.42
CD986361	GB|AP003561 ankyrin-like protein {Oryza sativa}	76%	0.10	0.03	0.29	0.13
CD990067	UP|C98A1_SORBI (O48956) Cytochrome P450 98A1	100%	0.21	0.07	0.43	0.17
CF031839	Zea mays clone Contig483.F mRNA sequence		0.17	0.07	0.30	0.12
CF062473	UP|Q8LBV7_ARATH plastid ribosomal protein L19	33%	0.15	0.07	0.29	0.17

^a ^GenBank accession number.

^b ^Annotation of each gene sequence was taken from the TIGR Maize Gene Index [12].

**Table 2:** 20 flint inbred lines each, out of a population of 270 recombinant inbred lines with the lowest or highest dNDF values, respectively.

**Low quality lines**	**dNDF**^a ^**(%)**	**High quality lines**	**dNDF (%)**
L01	45.21	H01	60.87
L02	50.07	H02	60.97
L03	50.41	H03	61.03
L04	50.72	H04	61.11
L05	51.37	H05	61.15
L06	51.37	H06	61.24
L07	51.80	H07	61.28
L08	52.55	H08	61.42
L09	52.78	H09	61.85
L10	52.78	H10	61.86
L11	53.46	H11	61.99
L12	53.47	H12	62.10
L13	53.72	H13	62.44
L14	53.75	H14	62.78
L15	53.77	H15	62.87
L16	53.82	H16	63.38
L17	53.86	H17	63.52
L18	53.94	H18	63.99
L19	53.94	H19	64.96
L20	53.95	H20	65.22
			
Mean	52.34	Mean	62.30
SD	2.12	SD	1.32

^a ^the percentage of digestible neutral detergent fiber.

## References

[B1] Ralph J, Guillaumie S, Grabber JH, Lapierre C, Barriere Y (2004). Genetic and molecular basis of grass cell-wall biosynthesis and degradability. III. Towards a forage grass ideotype. Comptes Rendus Biologies.

[B2] Barriere Y, Guillet C, Goffner D, Pichon M (2003). Genetic variation and breeding strategies for improved cell wall digestibility in annual forage crops. A review. Animal Research.

[B3] Gardiner JM, Coe EH, Melia-Hancock S, Hoisington DA, Chao S (1993). Development of a core RFLP map in maize using an immortalized F2 population. Genetics.

[B4] Barriere Y, Argillier O (1993). Brown-Midrib Genes of Maize – a Review. Agronomie.

[B5] Shi C, Koch G, Ouzunova M, Wenzel G, Zein I, Lubberstedt T (2006). Comparison of maize brown-midrib isogenic lines by cellular UV-microspectrophotometry and comparative transcript profiling. Plant Molecular Biology.

[B6] Borevitz JO, Chory J (2004). Genomics tools for QTL analysis and gene discovery. Curr Opin Plant Biol.

[B7] Jansen RC, Nap JP (2001). Genetical genomics: the added value from segregation. Trends Genet.

[B8] Li J, Burmeister M (2005). Genetical genomics: combining genetics with gene expression analysis. Human Molecular Genetics.

[B9] de Koning DJ, Haley CS (2005). Genetical genomics in humans and model organisms. Trends Genet.

[B10] Schadt EE, Monks SA, Drake TA, Lusis AJ, Che N, Colinayo V, Ruff TG, Milligan SB, Lamb JR, Cavet G (2003). Genetics of gene expression surveyed in maize, mouse and man. Nature.

[B11] Kirst M, Myburg AA, De Leon JPG, Kirst ME, Scott J, Sederoff R (2004). Coordinated genetic regulation of growth and lignin revealed by quantitative trait locus analysis of cDNA microarray data in an interspecific backcross of eucalyptus. Plant Physiology.

[B12] TIGR Maize Gene Index. http://compbio.dfci.harvard.edu/tgi/plant.html.

[B13] The Maize GDB. http://www.maizegdb.org.

[B14] Maize Genome Mapping project. http://maize-mapping.plantgenomics.iastate.edu/.

[B15] Melchinger AE, Utz HF, Schon CC (1998). Quantitative trait locus (QTL) mapping using different testers and independent population samples in maize reveals low power of QTL detection and large bias in estimates of QTL effects. Genetics.

[B16] Brem RB, Kruglyak L (2005). The landscape of genetic complexity across 5,700 gene expression traits in yeast. Proceedings of the National Academy of Sciences of the United States of America.

[B17] Brem RB, Yvert G, Clinton R, Kruglyak L (2002). Genetic dissection of transcriptional regulation in budding yeast. Science.

[B18] Grabber JH (2005). How do lignin composition, structure, and cross-linking affect degradability? A review of cell wall model studies. Crop Science.

[B19] Cano-Delgado A, Penfield S, Smith C, Catley M, Bevan M (2003). Reduced cellulose synthesis invokes lignification and defense responses in Arabidopsis thaliana. Plant Journal.

[B20] Schnable PS, Hochholdinger F, Nakazono M (2004). Global expression profiling applied to plant development. Curr Opin Plant Biol.

[B21] Salvi S, Tuberosa R (2005). To clone or not to clone plant QTLs: present and future challenges. Trends in Plant Science.

[B22] Hubner N, Wallace CA, Zimdahl H, Petretto E, Schulz H, Maciver F, Mueller M, Hummel O, Monti J, Zidek V (2005). Integrated transcriptional profiling and linkage analysis for identification of genes underlying disease. Nat Genet.

[B23] Sladek R, Hudson TJ (2006). Elucidating cis- and trans-regulatory variation using genetical genomics. Trends Genet.

[B24] Doss S, Schadt EE, Drake TA, Lusis AJ (2005). Cis-acting expression quantitative trait loci in mice. Genome Res.

[B25] Gibson G, Weir B (2005). The quantitative genetics of transcription. Trends Genet.

[B26] Corn production. http://maize.agron.iastate.edu/corngrows.html#stages.

[B27] Maize chip. http://www.plantgenomics.iastate.edu/maizechip/.

[B28] Hegde P, Qi R, Abernathy K, Gay C, Dharap S, Gaspard R, Hughes JE, Snesrud E, Lee N, Quackenbush J (2000). A concise guide to cDNA microarray analysis. Biotechniques.

[B29] Saeed AI, Sharov V, White J, Li J, Liang W, Bhagabati N, Braisted J, Klapa M, Currier T, Thiagarajan M (2003). TM4: a free, open-source system for microarray data management and analysis. Biotechniques.

[B30] The Iowa Schnable Lab. http://schnablelab.plantgenomics.iastate.edu/research/genomics/htp_est/ordering.php.

[B31] The Arizona BAC/EST resource center. http://genome.arizona.edu/orders/.

[B32] Churchill GA (2002). Fundamentals of experimental design for cDNA microarrays. Nature Genetics.

[B33] Eisen MB, Spellman PT, Brown PO, Botstein D (1998). Cluster analysis and display of genome-wide expression patterns. Proc Natl Acad Sci U S A.

[B34] Pan W (2002). A comparative review of statistical methods for discovering differentially expressed genes in replicated microarray experiments. Bioinformatics.

[B35] Ooijen V, W. J, Boer MP, Jansen RC, Maliepaard C (2002). MapQTL 4.0, Software for the caculation of QTL position on genetic maps.

[B36] Churchill GA, Doerge RW (1994). Empirical Threshold Values for Quantitative Trait Mapping. Genetics.

[B37] Doerge RW, Churchill GA (1996). Permutation tests for multiple loci affecting a quantitative character. Genetics.

[B38] ArrayExpress. http://www.ebi.ac.uk/arrayexpress.

